# Termite mound architecture regulates nest temperature and correlates with species identities of symbiotic fungi

**DOI:** 10.7717/peerj.6237

**Published:** 2019-01-16

**Authors:** Risto Vesala, Anni Harjuntausta, Anu Hakkarainen, Petri Rönnholm, Petri Pellikka, Jouko Rikkinen

**Affiliations:** 1 Finnish Museum of Natural History, Botany Unit, University of Helsinki, Helsinki, Finland; 2 Organismal and Evolutionary Biology Research Programme, Faculty of Biological and Environmental Sciences, University of Helsinki, Helsinki, Finland; 3 Department of Built Environment, Aalto University, Espoo, Finland; 4 College of Global Change and Earth System Science, Beijing Normal University, Beijing, China; 5 Earth Change Observation Laboratory, Department of Geosciences and Geography, University of Helsinki, Helsinki, Finland

**Keywords:** *Macrotermes*, *Termitomyces*, Fungus-growing termites, Basidiomycota, Thermoregulation, Mound building, Symbiont diversity, Photogrammetry, 3D modeling

## Abstract

**Background:**

Large and complex mounds built by termites of the genus *Macrotermes* characterize many dry African landscapes, including the savannas, bushlands, and dry forests of the Tsavo Ecosystem in southern Kenya. The termites live in obligate symbiosis with filamentous fungi of the genus *Termitomyces*. The insects collect dead plant material from their environment and deposit it into their nests where indigestible cell wall compounds are effectively decomposed by the fungus. Above-ground mounds are built to enhance nest ventilation and to maintain nest interior microclimates favorable for fungal growth.

**Objectives:**

In Tsavo Ecosystem two *Macrotermes* species associate with three different *Termitomyces* symbionts, always with a monoculture of one fungal species within each termite nest. As mound architecture differs considerably both between and within termite species we explored potential relationships between nest thermoregulatory strategies and species identity of fungal symbionts.

**Methods:**

External dimensions were measured from 164 *Macrotermes* mounds and the cultivated *Termitomyces* species were identified by sequencing internal transcribed spacer (ITS) region of ribosomal DNA. We also recorded the annual temperature regimes of several termite mounds to determine relations between mound architecture and nest temperatures during different seasons.

**Results:**

Mound architecture had a major effect on nest temperatures. Relatively cool temperatures were always recorded from large mounds with open ventilation systems, while the internal temperatures of mounds with closed ventilation systems and small mounds with open ventilation systems were consistently higher. The distribution of the three fungal symbionts in different mounds was not random, with one fungal species confined to “hot nests.”

**Conclusions:**

Our results indicate that different *Termitomyces* species have different temperature requirements, and that one of the cultivated species is relatively intolerant of low temperatures. The dominant *Macrotermes* species in our study area can clearly modify its mound architecture to meet the thermal requirements of several different symbionts. However, a treacherous balance seems to exist between symbiont identity and mound architecture, as the maintenance of the thermophilic fungal species obviously requires reduced mound architecture that, in turn, leads to inadequate gas exchange. Hence, our study concludes that while the limited ventilation capacity of small mounds sets strict limits to insect colony growth, in this case, improving nest ventilation would invariable lead to excessively low nest temperatures, with negative consequences to the symbiotic fungus.

## Introduction

Fungus-growing termites of the genus *Macrotermes* build large and conspicuous mounds that characterize many savanna landscapes in Africa. Mounds, built above the subterranean termite nests, ensure insulation and protection against environmental fluctuations and predators (Bonabeau et al., 1998; Noirot & Darlington, 2000; Korb, 2011). The mound helps to generate a specific nest microclimate by regulating temperature and humidity (Korb, 2003, 2011). Mound structures also play a major role in gas exchange: large nests must be effectively ventilated to remove the excess CO_2_ and heat generated by the metabolism of the termites and the fungal symbiont that is cultivated in specialized chambers within the nests (Weir, 1973; Korb & Linsenmair, 1999a; Korb, 2003).

Two closely related species of *Macrotermes* occur sympatrically in southern Kenya (Bagine, Brandl & Kaib, 1994; Pomeroy, 2005; Vesala et al., 2017). The species can be easily identified by mound architecture, the most obvious difference being in ventilation systems: *Macrotermes subhyalinus* builds mounds with open and *M. michaelseni* mounds with closed ventilation systems (Darlington, 1984, 1985; Bagine, Brandl & Kaib, 1994; Vesala et al., 2017).

Open mounds of *M. subhyalinus* are equipped with funnel-like large ventilation shafts that open to the mound surface. Variably shaped openings located at different elevations promote a wind-induced Venturi effect, leading to a rapid flow of air through the mound (Weir, 1973). Air is typically drawn out from large centrally situated openings on the mound top and sucked in from peripheral and basal funnels (Weir, 1973; Korb, 2011). The air passages do not ventilate the nest or fungus gardens directly but are separated from them by soil layers (Darlington, 1984; Korb, 2011).

Closed mounds of *M. michaelseni* lack open ventilation shafts and air circulation within a mound is mainly driven by a temperature gradient (Korb, 2011; Ocko et al., 2017). During sunny days air from the nest and fungus gardens flows upwards in narrow cavities near the mound surface which is effectively heated by the sun (Korb, 2011; Ocko et al., 2017). Gas exchange takes place on the mound top through the porous outer surface induced by diffusion and wind (Turner, 2001; Korb, 2011; Ocko et al., 2017). Fresh air is supplied to the nest via a large central shaft. During night the mound surface cools and the direction of the air flow is reversed (Ocko et al., 2017).

In addition to major differences in the ventilation systems produced by different termite species, there is also intraspecific variation in mound architecture. Such variation has been studied in the Ivory Coast where *M. bellicosus* builds closed mounds quite similar to those of *M. michaelseni.* The mounds in open shrub savanna had many ridges and turrets and exhibited a more complex architecture than the relatively simple mounds in shady gallery forests (Korb & Linsenmair, 1998a). When the internal temperatures of simple nests in the forest were experimentally increased the termites responded by adding architectural features that increased mound complexity, thus demonstrating that termites can actively change mound architecture to regulate nest temperatures (Korb & Linsenmair, 1998b). In the relatively cool gallery forest the termites were able to maintain appropriate nest temperatures by building compact dome-like mounds. However, on the basis of elevated CO_2_ levels the reduced mounds had limited capacity to facilitate effective gas exchange (Korb & Linsenmair, 1999a). Also the reproductive success of compact mounds was lower than that of the complex mounds (Korb & Linsenmair, 1999b).

The mound building behavior of East African *Macrotermes* species have not yet been extensively studied. Closed mounds of Kenyan *M. michaelseni* are known to vary from steeple or dome shaped simple cones to wide and complex many-turreted mounds. Open mounds of *M. subhyalinus* range from small and simple mounds to large and monumental ones that can be several meters high. Often the large mounds have elevated ventilation shafts which further increase architectural diversity.

A central objective of this study was, for the first time, to incorporate data on the diversity of fungal symbionts into the research of termite mound architecture. Previous studies have showed that each colony of fungus-growing termites always cultivates only one heterokaryotic strain of *Termitomyces* as a monoculture (Aanen et al., 2002, 2009; Katoh et al., 2002; De Fine Licht, Andersen & Aanen, 2005; Moriya et al., 2005; Makonde et al., 2013). In most *Macrotermes* species, including *M. subhyalinus* and *M. michaelseni*, the symbiotic fungus is thought to be horizontally transmitted, meaning that a newly established young termite colony acquires its *Termitomyces* symbiont from the nest surroundings, presumably as fungal spores (Korb & Aanen, 2003). The mechanisms in which a certain fungal species is selected for cultivation remain largely unknown.

*Macrotermes* termites are known to have their own set of *Termitomyces* species that is generally shared between different species within the genus but not with other genera of fungus-growing termites (Aanen et al., 2002; Rouland-Lefèvre et al., 2002; Frøslev et al., 2003; Osiemo et al., 2010; Nobre et al., 2011; Makonde et al., 2013). Our previous study in the Tsavo ecosystem demonstrated that *M. subhyalinus* and *M. michaelseni* in that area cultivated three *Termitomyces* species, and two of these fungi were found from nests of both termite species (Vesala et al., 2017). The highly uneven distribution of the three *Termitomyces* species across different habitats suggested that there are unknown ecological differences between these symbionts (Vesala et al., 2017).

As a work hypothesis for this study we proposed that the three *Termitomyces* species might have different temperature requirements for metabolism and optimal growth. As the fungus-growing termites are known to use architectural modification of above-ground mounds to regulate nest interior temperatures (Korb & Linsenmair, 1998a, 1998b) we expected to also find links between mound architecture and cultivated *Termitomyces* species. To determine such potenital relationships, we measured the height and basal width of 164 *M. subhyalinus* and *M. michaelseni* mounds with known fungal symbionts. We also compared internal nest temperatures of closed *M. michaelseni* and open *M. subhyalinus* mounds. Finally, to get detailed information of the thermoregulatory effects of intraspecific architectural variation we recorded annual temperature regimes of 14 different-sized open *M. subhyalinus* mounds. The architectural features of these mounds were studied by producing photogrammetric 3D models.

## Material and Methods

### Research area and studied termite mounds

**Figure 1:**
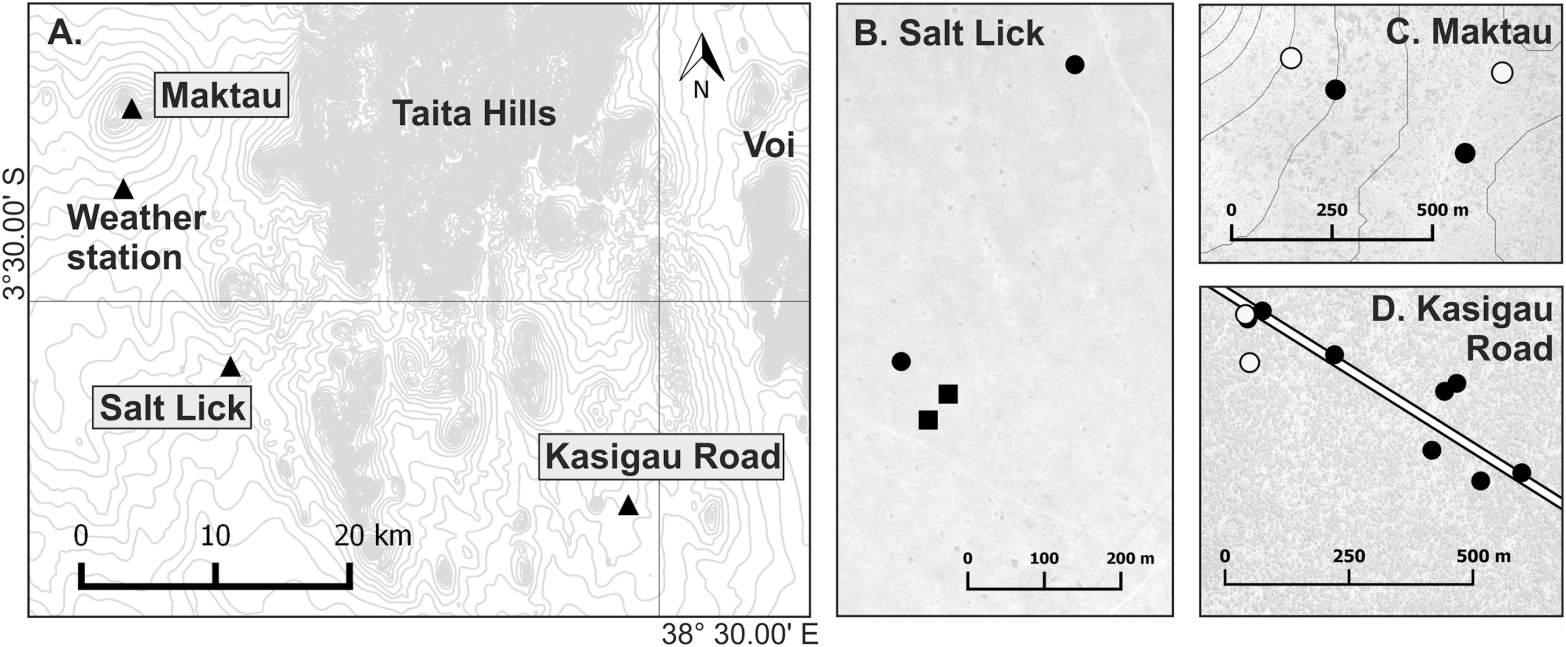
Map of the research area. (A) Location of the three study sites: Maktau (3°22′14″S, 38°8′41″E), Salt Lick (3°32′37″S, 38°12′43″E), and Kasigau Road (3°38′14″S, 38°28′34″E). Small-scale maps (B–D) show locations of the studied termite mounds within the sites. Circle = *Macrotermes subhyalinus*; Square = *Macrotermes michaelseni*; Solid symbol = *Termitomyces* sp. A; Open symbol = *Termitomyces* sp. C. Contour interval 20 m.

Field work was performed in Taita–Taveta County, southern Kenya, under the research authorization from National Commission for Science, Technology and Innovation of Kenya (NACOSTI/P/17/54522/15694). A total of 164 *Macrotermes* mounds were selected from several locations within maximum distance of 80 km. All mounds with an open ventilation system were considered to house *M. subhyalinus* colonies and those with closed ventilation systems *M. michaelseni* colonies. Temperature measurements were conducted in 18 of these termite mounds at three different study sites (Table 1; Fig. 1): Kasigau Road (*Commiphora* woodland, elevation 815 m.a.s.l.), Salt Lick (grassland, elevation 890 m.a.s.l.), and Maktau Hills (semi-open bushland, elevation 1,215–1,270 m.a.s.l.). For more information on the study sites, see Vesala et al. (2017). Temperature measurements were performed during two campaigns: January–August 2015 in two *M. subhyalinus* and two *M. michaelseni* mounds at Salt Lick (first campaign), and March 2016–April 2017 in 14 different-sized *M. subhyalinus* mounds at Kasigau Road and Maktau (second campaign; Fig. 1; Table 1).

**Table 1:** Measured data and *Termitomyces* species of all termite mounds from which temperature data was obtained during the study.

Colony name	Study site	Mound type	*Termitomyces* species	Height^a^ (m)	Mean basal width (m)	Volume (m^3^)	Surface area (m^2^)	Number of shafts	Maximum distance of shafts (m)	Maximum width of shafts (m)	Miller’s LAI^b^
Studied mounds in Salt Lick 2015											
TS18	Salt Lick	Closed	A	1.20	3.5	–	–	–	–	–	–
TS19	Salt Lick	Closed	A	1.40	4.0	–	–	–	–	–	–
TS14	Salt Lick	Open (large)	A	1.25	3.7	–	–	–	–	–	–
TS56	Salt Lick	Open (large)	A	1.20	3.2	–	–	–	–	–	–
Studied mounds in Kasigau Road and Maktau 2016–2017											
TM36	Maktau	Open (miniature)	C	0.25	1.4	0.13	2.64	10	1.3	0.08	0.04
TR09	Kasigau Road	Open (miniature)	C	0.20	1.3	0.10	2.26	6	0.8	0.11	1.07
TR172	Kasigau Road	Open (miniature)	C	0.10	0.8	0.01	0.64	8	0.8	0.12	0.71
TR182	Kasigau Road	Open (miniature)	A	0.00	0.9	0.03	1.03	5	0.6	0.08	1.26
TR184	Kasigau Road	Open (miniature)	A	0.15	1.1	0.02	1.69	8	0.9	0.08	0.80
TR185	Kasigau Road	Open (miniature)	A	0.15	0.8	0.01	0.43	7	0.7	0.08	0.88
TM08	Maktau	Open (large)	C	0.55	1.7	0.68	5.74	16	2.2	0.20	0.39
TM10	Maktau	Open (large)	A	1.55	3.0	4.29	17.43	34	3.6	0.23	0.00
TM14	Maktau	Open (large)	A	1.20	3.3	4.46	18.04	25	3.6	0.18	0.67
TR10	Kasigau Road	Open (large)	A	0.24	3.1	1.03	11.42	30	3.1	0.35	0.61
TR83	Kasigau Road	Open (large)	A	1.40	2.3	2.44	15.60	23	2.2	0.17	0.87
TR101	Kasigau Road	Open (large)	A	1.00	3.1	3.78	18.85	23	2.9	0.38	0.69
TR109	Kasigau Road	Open (large)	A	1.75	3.1	6.08	24.46	25	2.6	0.31	0.33
TR161	Kasigau Road	Open (large)	A	0.30	1.9	0.34	4.28	26	2.1	0.20	0.14

**Notes:**

a Measured from soil surface to the top of mound body, higher turrets not included.

b Mean value measured from five locations around the mound.

*Termitomyces* symbionts in all 164 studied termite colonies were identified on the basis of DNA extracted from fungal nodules collected from fungus chambers. ITS1-5.8S-ITS2 region was amplified using direct PCR method (Phire Plant Direct PCR Kit; Thermo Scientific, Waltham, MA, USA) with the primer pair ITS1FT (Aanen et al., 2007) and ITS4 (White et al., 1990). Fungal species were identified based on either comparison of complete sequences or the two polymorphic sites within ITS1 similarly as described in Vesala et al. (2017). Some of the colonies were the same that were identified during our previous studies (Vesala et al., 2017). For a list of all 164 colonies included in this study and GenBank accession numbers for published sequences, see Table S1. Accession numbers for the novel ITS sequences produced in this study are MK275596–MK275616.

### Temperature measurements

Air and soil temperature data were obtained from a weather station close to the Maktau site (elevation 1,070 m.a.s.l.; Fig. 1). In order to evaluate the extent of regional temperature differences induced by elevation and local vegetation short-term measurements of air temperature were also performed at all three study sites.

Data on nest interior temperatures and ambient air temperatures at the study sites were obtained with small temperature data loggers (iButton Thermochron DS1922L; Maxim, San Jose, CA, USA). The target mounds were opened by digging carefully from the mound base on western side and a sensor was installed into the first active fungus chamber encountered. After installation the chamber wall was repaired with soil clumps and the remaining hollow was filled with loose soil up to its original level. Before installation the sensors were wrapped in several layers of fine iron net in order to prevent termite damage. To mark the location of sensors thin iron wire leading to the mound surface was attached to each sensor. The data loggers measuring local ambient air temperature were attached to tree trunks at height of approximately two m from the ground level and covered by light-impermeable plastic shields to shade them from direct solar radiation.

During the first measurement campaign (January–August 2015 in two *M. michaelseni* and two *M. subhyalinus* mounds at Salt Lick) data loggers were programmed to record the temperature at each full hour (00:00, 01:00, 02:00, etc.). Because of the limited memory capacity of the devices, the nests had to be opened and data retrieved once in April 2015, after which the sensors were immediately returned to the same chamber. One of the data loggers malfunctioned during the second half of the campaign and, due to this, data for the rest of the period was obtained only from two closed (TS18, TS19) and one open mound (TS56).

During the second campaign (March 2016–April 2017 in 14 *M. subhyalinus* mounds) data loggers recorded the temperature at 3 h intervals (00:00, 03:00, 06:00, 09:00, 12:00, 15:00, 18:00, and 21:00). This allowed the devices to remain in the mounds for a full year without interruptions. Two termite colonies (TM08 and TR185) died during the second measurement campaign. In all other cases the sensors remained in the close proximity of an active fungus comb for the entire campaign, though in most cases, the termites had covered the data loggers in soil and sealed them into the closest chamber wall.

### Termite mound architecture

**Figure 2:**
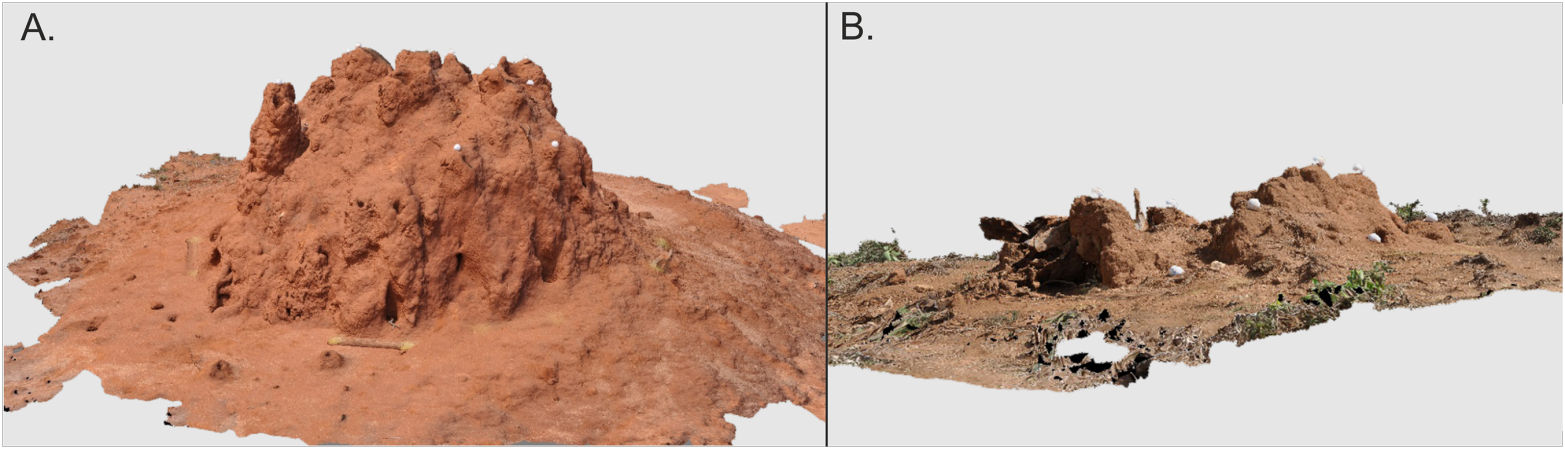
3D models of two *Macrotermes subhyalinus* mounds. (A) Mound TM10 represents a typical large mound (height 1.55 m, mean basal width 3.0 m). (B) TM36 was a small miniature mound (height 0.25 m, mean basal width 1.4 m). For more details, see Table 1. The spherical objects on the mounds are golf balls (diameter 4.2 cm) which were used to confirm successful image orientation in photogrammetric processing. Images by Petri Rönnholm.

The height and basal width (in two cardinal directions: N–S, W–E) of all 164 studied mounds were measured in field. Height was always measured from the ground level to the top of mound body, higher turrets or chimneys (if present) were not included. To obtain more detailed architectural data from the 14 *M. subhyalinus* mounds studied during the second campaign (Table 1), digital images were taken from different positions around each mound. Depending on mound size a total of 50–120 images from distance of two to five m from the mound were acquired with Nikon d5000 and a 35 mm fixed focal length lens. Half of the images were taken from the height of ca. 50 cm and half from the height of ca. 200 cm from the ground level. Images were combined into 3D-models (Fig. 2) by using Agisoft PhotoScan software. The scales of the models were corrected by placing a scale bar with known length in the scene during the image acquisition. Even if the image orientation process was automatic, we placed 10 small spherical targets with the diameter of 42 mm to the scene in order to enable manual measurements. These targets became essential only in one case, in which automation could not connect all images into the same block. Volumes and areas of the final 3D models were computed by using Geomagic Qualify 11 software. Widths of the ventilation shafts and their mutual distances were measured from the produced orthophotographs by using Fiji ImageJ (v. 1.51) software.

To obtain information about the size and structure of the belowground nests of especially small *M. subhyalinus* mounds (“miniature mounds”, see results) two of these colonies (TR09 and TR183) were excavated. The maximum width between the outernmost fungus combs and the total height of nest interior were measured from both colonies.

### Canopy cover

Canopy cover over the 14 *M. subhyalinus* mounds of the second campaign (March 2016–April 2017) was evaluated by determining leaf area index (LAI) from digital hemispheric photographs taken with Nikon D700 equipped with Sigma 8 mm 1:3.5 EX DG. The photographs were taken directly upward from the mound top and from spots three m from the mound center toward each cardinal direction. The images were acquired from the height of one m except the images that were taken from the top of mounds higher than that Miller’s (1967) LAI was calculated for the images using Hemisfer software (Schleppi et al., 2007; Thimonier, Sedivy & Schleppi, 2010). Mean of the five values obtained for each mound was used to quantify the level of canopy coverage.

### Statistical analyses

Statistical analysis were performed in RStudio version 1.0.153, (RStudio Team, 2016). To see whether significant temperature differences existed between the study sites we compared air temperatures measured at the weather station to those measured at Kasigau Road and Maktau study sites during the second measurement campaign by using simple linear regression. Diurnal mean temperatures of 234 days at Kasigau Road and 194 days in Maktau and corresponding mean temperatures measured simultaneously at the weather station were used in the analysis.

To study the nest interior temperatures of the 14 *M. subhyalinus* mounds measured during the second campaign we first calculated diurnal mean temperatures from the full data. The warmest and the coolest months of the year (March and July) were then selected for more detailed analysis. Data from the two colonies that perished during the survey (TR185 and TM08) was omitted, as the temperature regimes of these nests were obviously disrupted. Initial data exploration showed that the nest interior temperature was clearly affected by both ambient air temperature and mound size, with small mounds generally having higher temperatures than large ones.

To further elucidate factors that affected nest temperatures we first built a generalized least squares model (GLS in nlme package, Pinheiro et al., 2017) with the nest diurnal mean temperature as the dependent variable. Data for the two months (March and July) were analyzed seperately to identify potential differences in nest thermoregulation mechanisms between the warm and cool season. We first included ambient air temperature (measured at the weather station) as the only fixed variable. Diurnal mean temperature of the previous day was used because of systematic lag between air temperatures and nest interior temperatures. As the data included several repeated measurements from each studied colony, temporal autocorrelation was included in the models by using AR(1) correlation structure with day as time covariate and the colony as a grouping factor. To elucidate the importance of different mound architectural features (height, width, volume, area, distance between ventilation shafts, and mound type: “miniature” vs. “large”) for thermoregulation of fungal chambers, we nested each variable into the initial model, one at a time. Each of the parallel improved models were compared with the initial model by using likelihood ratio tests and Akaike information criterion (AIC). Maximum likelihood estimation was used to fit the models, as required for meaningful fixed effects comparisons (Zuur et al., 2009).

To test other potentially significant variables, we used stepwise model selection to include LAI, study site and all relevant interaction terms as additional fixed variables to the model. The model with ambient air temperature and the best architectural predictor (mound type) was selected for this further improvement. Non-significant terms at the level of 0.05 were dropped. Final models were fitted using restricted maximum likelihood (REML) for validation. Residuals of the models including all significant variables and interactions were normally distributed and didn’t show any particular patterns when plotted against fitted values.

Distribution of the three *Termitomyces* species (A, B, and C) in different-sized *Macrotermes* mounds was studied by using one-way ANOVA. The data set included size measurements (height and mean basal width) and *Termitomyces* species from all 164 termite mounds. Analysis was done separately for the two variables (height and width) and for the two *Macrotermes* species.

## Results

### Air and soil temperatures

**Figure 3:**
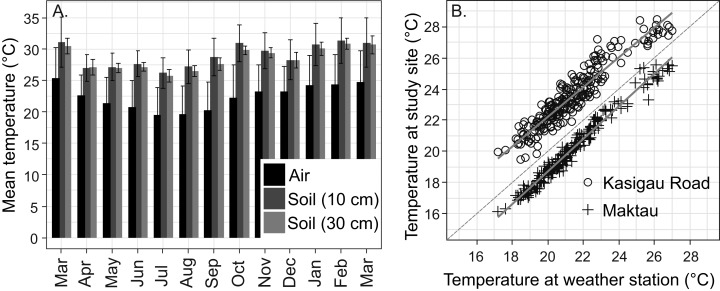
Temperature data from the study area. (A) Annual variation in air and soil temperatures (mean with standard deviation) at the Maktau weather station during the second measurement campaign (March 2016–March 2017). (B) Linear regressions and data points between the daily mean temperatures at the Maktau weather station and two field sites (Kasigau Road and Maktau) during 1.3.2016–26.10.2016. Salt Lick was not included in this comparison because the local temperature measurements at the site were performed during previous year (2015).

The average annual air temperature at the Maktau weather station for March 2016–February 2017 (the second measurement campaign) was +22.3 °C. The highest temperatures were measured during the short dry season in March (mean +25.4 °C, SD 4.9 °C) and the lowest soon after the long rains in July (mean +19.5 °C, SD 4.4 °C; Fig. 3A). The general pattern of monthly temperatures at the weather station during the first measurement campaign (2015) was quite comparable with those measured in 2016–2017.

The soil temperatures at the weather station exhibited a similar annual pattern as the air temperatures, but remained consistently higher (Fig. 3A). The annual mean temperature at the depth of 30 cm was slightly lower and showed less variation (+28.4 °C, SD 1.9 °C) than the annual mean temperature at the depth of 10 cm (+28.8 °C, SD 3.4 °C).

Daily mean temperatures measured at the Kasigau Road and Maktau field sites during the second campaign exhibited a similar annual pattern as those measured at the weather station (linear regression; Kasigau Road: coefficient = 0.947, *R*^2^ = 0.886, *p* < 0.001; Maktau: coefficient = 1.043, *R*^2^ = 0.974, *p* < 0.001, Fig. 3B). Temperatures at the Kasigau Road site were on average 2 °C higher and at the Maktau site on average 1 °C lower than those measured simultaneously at the weather station (Fig. 3B).

### Comparison of nest temperatures of open and closed mounds

**Figure 4:**
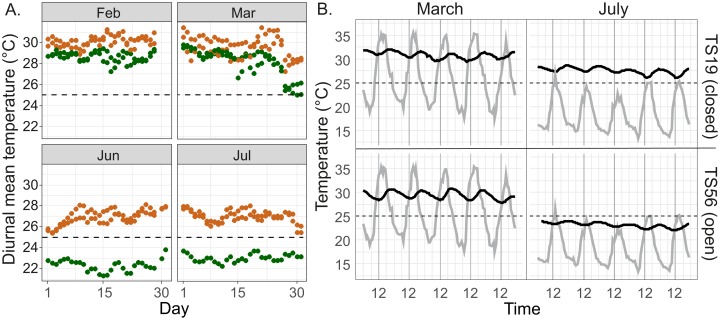
Temperature regimes in closed and open *Macrotermes* mounds. (A) Diurnal mean temperatures in two open (TS14, TS56; green) and two closed (TS18, TS19; orange) termite mounds during the two warmest months (February, March) and two coolest months (June, July) of the year. (B) Example of diurnal variations of nest internal temperature in closed mound (TS19, upper) and open mound (TS56, lower) during the warmest and coolest month (1.–5.3.2015 and 1.–5.7.2015). Gray line = ambient air temperature; Black line = temperature inside fungus chamber of termite nest. Air temperatures are based on local measurements at the field site.

Measurements at the Salt Lick site in January–August 2015 (campaign 1) revealed that interior temperatures in closed *M. michaelseni* mounds were higher than those in open *M. subhyalinus* mounds on most days. The highest nest temperatures were measured during the period from February to March. During this period diurnal mean temperatures in both mound types remained above 27 °C on most days. The two closed mounds had higher mean temperatures than the two open mounds during all days in February and most days in March (Fig. 4A). The lowest nest interior temperatures were measured in June and July. During this period the diurnal mean temperatures in two closed mounds ranged from 25 to 28 °C, whereas the temperatures measured from the open mound (TS56) were constantly several degrees lower (21–24 °C; Fig. 4A).

Ambient air temperatures were always highest in the early afternoon (12:00–15:00) and lowest just before sunrise (Fig. 4B). However, inside the fungus chambers the highest temperatures were recoded between 18:00–24:00 and the lowest temperatures around midday. The internal temperature of termite mounds tracked ambient air temperatures in a way that temperature in fungus chambers slightly increased after warm and decreased after cool days (see example in Fig. 4B, July). Patterns of diurnal variation in mound temperatures was similar in open and closed mounds, with the amplitude of variation always remaining within 0–4 °C of the average even while concurrent variation in ambient air temperatures could be more than 20 °C during the warmest season (Fig. 4B).

### Architectural variation and nest temperatures in open mounds

On the basis of size and internal structure we classified the 14 open *M. subhyalinus* mounds measured during the second campaign (March 2016–April 2017) into two categories: eight “large” mounds and six small “miniature” mounds. All miniature mounds were less than 1.5 m wide, and had only a few narrow ventilation shafts (diameter < 12 cm) within a maximum distance of 1.3 m between the outermost shafts (Table 1). The large mounds always had more than 15 wide ventilation shafts (diameter up to 38 cm) and the distance between the outermost shafts was always at least two m (Table 1). Also the internal nest structure of the two mound categories was different. In two excavated miniature mounds (TR09, TR183) total height from the uppermost fungus combs to the nest bottom was less than 55 cm and maximum width between the outernmost fungus combs was less than 100 cm. All the fungus gardens in these nests were very close together immediately surrounding the queen chamber and larval nurseries. The fungus combs of large mounds were typically situated in separate fungus chambers (with partitions) and were generally distributed over a much wider area. The structure of miniature mounds was quite uniform, while large mounds exhibited considerable variation in architectural features including height, width, volume, surface area, and the shape and number of ventilation shafts.

Comparison of several generalized least squares (GLS) models each with different mound architectural features nested in the initial model (ambient air temperature as the only fixed variable) showed that factorial classification into the mound categories “large” vs. “miniature” produced the greatest improvement to model fit (Table S2). This classification also produced the lowest AIC values both in March and July. The second and third best predictors of nest temperature were maximum distance of ventilation shafts and mound basal width, respectively, which both improved model fit much more than height, volume, or surface area (Table S2). Model improvement by architectural variables in general was much more significant in July than in March (Table S2).

**Figure 5:**
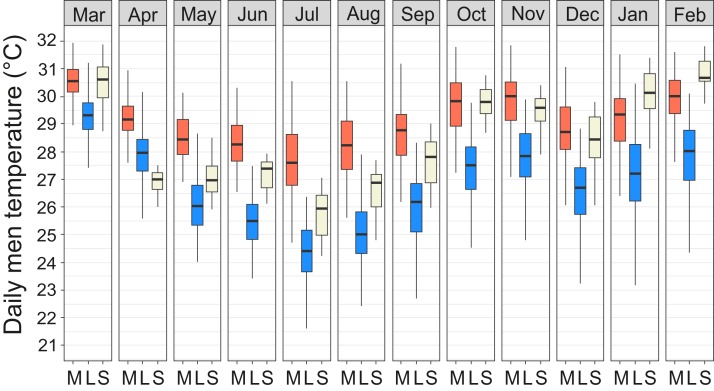
Temperature regimes in miniature and large *Macrotermes subhyalinus* mounds. Variation of daily mean temperatures in miniature (M) and large (L) mounds and in soil at depth of 30 cm (S) during different months (March 2016–February 2017). Data include measurements only from active nests.

Year-round temperature measurements showed that the miniature mounds were consistently warmer throughout the year and the difference was most pronounced during the cool season from June to August (Fig. 5). The median of the diurnal mean temperatures for the coolest and warmest months (July/March) were 27.7/30.6 °C in miniature mounds and 24.3/29.3 °C in large mounds, respectively. When compared to the soil temperatures measured at the weather station, miniature mounds were on average warmer and large mounds on average cooler than normal soil at the depth of 30 cm (Fig. 5). The only exceptions were recorded between January and March when temperatures in miniature mounds were slightly cooler, and April when temperatures in large mounds were slightly warmer than the average soil temperature at the weather station (Fig. 5).

Further stepwise improvement of the GLS models by adding the variables LAI and study site produced different results in March and July. In March study site (Kasigau Road vs. Maktau) had a significant effect on nest temperatures (coefficient = 3.9, *t* = 3.21, *p* = 0.0014) together with ambient air temperature and mound type. Also interaction terms of ambient air temperature with both mound type and study site were significant (Table S3). Canopy cover (LAI) did not have a significant effect on nest temperatures in March. In July the effect of LAI was significant (coefficient = 1.3, *t* = 2.2, *p* = 0.0271) together with the variables ambient air temperature and mound type (Table S3). Conversely, study site did not have a significant effect on nest temperatures in July. Neither were any of the interactions tested significant.

### Distribution of *Termitomyces* species in relation to mound architecture

**Figure 6:**
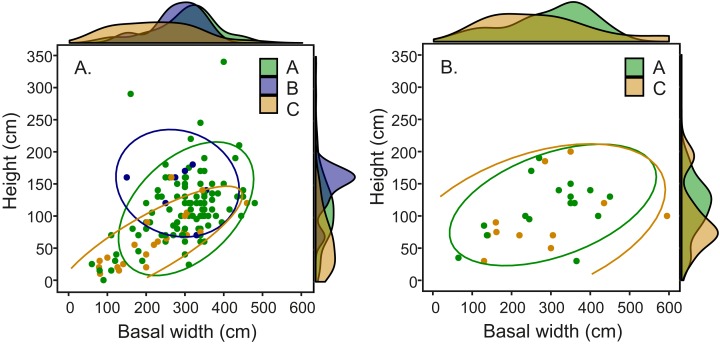
Mound dimensions (height and basal mean width) and fungal identity in (A) open *M. subhyalinus* and (B) closed *M. michaelseni* mounds. Ellipses represent 95% confidence interval for dimensions of mounds where each *Termitomyces* species was cultivated.

Comparison of fungal symbiont identity and mound architecture in the 164 *Macrotermes* mounds revealed that *M. subhyalinus* mounds with *Termitomyces* sp. C were significantly smaller (ANOVA: *p* < 0.001 for both height and width) than the mounds with either *Termitomyces* sp. A or B (Table S4). As clearly shown in Fig. 6A species C was common in small miniature mounds of *M. subhyalinus* but rarely found from large open mounds. A similar relationship was not found from closed *M. michaelseni* mounds (Fig. 6B; Table S4). *Termitomyces* sp. B was only found from relatively few large *M. subhyalinus* mounds, and was absent from all closed *M. michaelseni* mounds (Fig. 6).

Either *Termitomyces* sp. A or *Termitomyces* sp. C was present in all *Macrotermes* mounds from which temperature data was obtained (Table 1). Most of these nests had *Termitomyces* sp. A with *Termitomyces* sp. C being housed only in three miniature mounds (TM36, TR09, TR172), and one large *M. subhyalinus* mound (TM08) that perished during the survey. Three of the 10 *M. subhyalinus* mounds with *Termitomyces* sp. A were miniature mounds (TR182, TR184, and TR185), and also one of these colonies (TR185) perished during the course of the survey.

## Discussion

### Temperatures in open and closed *Macrotermes* mounds

Above-ground mound architecture allows termites to effectively regulate environmental conditions within the nest interior. Relationships between mound structures and nest temperatures and/or ventilation have been observed in several studies (Harris, 1956; Lüscher, 1961; Ruelle, 1964; Weir, 1973; Korb & Linsenmair, 1998a, 1998b, 1999a, 2000a, 2000b; Turner, 2001; Korb, 2003, 2011; King, Ocko & Mahadevan, 2015; Ocko et al., 2017). Previous studies have mostly focused on *M. bellicosus* which is a common and widely-distributed termite species especially in western and central Africa (Ruelle, 1970). It builds closed mounds that are functionally comparable to those built by *M. michaelseni*, although some minor differences exist (Korb, 2011). The East African species *M. subhyalinus* and *M. jeanneli*, which both build open mounds have so far received less attention.

During the first measurement campaign we compared nest temperature patterns within closed *M. michaelseni* mounds to those of open *M. subhyalinus* mounds located side by side in an open grassland savanna. The diurnal oscillations in nest temperatures were qualitatively similar in both nest types, but the temperatures remained constantly lower in the open mounds. The overall amplitude of diurnal temperature fluctuations varied somewhat between mounds (see Fig. 4B: July), but such differences were most likely due to the fact that termites typically covered the temperature sensors with layers of soil which probably had a slight effect on the recorded within-day temperature amplitudes. The 6–12 h delay in maximum and minimum temperatures between the fungus chambers and ambient air, recorded from both open and closed mounds, are comparable to those found by Korb & Linsenmair (1998a, 1998b, 2000a) from closed *M. bellicosus* mounds in the Ivory Coast.

On larger geographical scales ambient air temperatures tend to decline along with rising elevation. Thus, while the altitudinal ranges of *M. subhyalinus* and *M. michaelseni* overlap in our study area, the consistently lower temperatures of *M. subhyalinus* mounds may be related to its tendency to favor lower elevations and thus also hotter climates (Bagine, Brandl & Kaib, 1994; Pomeroy, 2005). Thus the open *Macrotermes* mounds with large ventilation shafts might represent an adaptation to the very high temperatures seasonally experienced in low-lying equatorial environments. However, open mounds are also constructed by some species of the termite genus *Odontotermes* that often live in shady and relatively cool forests (Korb, 2011).

### Architecture and nest temperatures of *M. subhyalinus* mounds

*Macrotermes subhyalinus* is the dominant mound builder both in open grasslands and dense woodlands in the Tsavo ecosystem (Vesala et al., 2017; Rikkinen & Vesala, 2017). As is the case with several other *Macrotermes* species, variation in the size and structural complexity of *M. subhyalinus* mounds is high, with mounds ranging from monumental, many-turreted castles to small miniature mounds. Our long-term temperature data logging revealed that structural differences between miniature and large mounds had a major effect on nest interior climate. Temperatures in miniature mounds remained constantly high throughout the measurement period, whereas temperatures in large *M. subhyalinus* mounds dropped several degrees during the cool season from June to August (Fig. 5).

Comparison of different architectural variables demonstrated that neither mound volume nor mound height were particularly good predictors of the interior temperatures of *M. subhyalinus* nests. Instead, small basal width and short distance between the outermost ventilation shafts were features which correlated with high nest temperatures. In two excavated miniature mounds the underground nests were relatively small and the fungus combs were tightly packed around the queen chambers and nurseries. In these colonies maintenance of nest temperatures at near 30 °C throughout the year was probably promoted by the placement of all fungal gardens and all resident termites into a very restricted space. For maintaining high nest temperatures this type of placement could even be compared to the “huddling behavior” of many other animals, including honey bees, emperor penguins, and naked mole rats (Withers & Jarvis, 1980; Kronenberg & Heller, 1982; Gilbert et al., 2006). Also a limited number of open air passages obviously helps in temperature maintenance. As canopy cover (LAI) also had a significant increasing impact on nest interior temperatures during cool season, the placement of miniature mounds under trees may also have acted to increase nest temperatures by preventing heat loss during cool nights.

The existence of specific class of “miniature mounds” appears to have been overlooked in previous literature. Such nests have probably been mainly interpreted as recently established and growing colonies (cf. Darlington, 1990). However, our repeated observations during a time span of over 4 years did not reveal any change in the height or width of some miniature mounds (TM36, TR09, and TR172), suggesting that they were not actively growing and eventually developing into large mounds. Thus, although in many cases small mounds must obviously represent transient phases in development toward larger mounds, in some cases the miniature architecture may be more or less permanent. In *M. subhyalinus* mounds the volume of air passing through the open ventilation system is proportional to the volume of the above-ground mound (Weir, 1973). The few narrow ventilation shafts of small miniature mounds cannot facilitate gas exchange required by very large colonies. Thus, termite colonies living in miniature mounds must always be relatively small.

### Distribution of *Termitomyces* species in different mounds

In our study area *Termitomyces* species C was the dominant fungal symbiont in constantly warm miniature mounds but rarely found from larger *M. subhyalinus* mounds in which *Termitomyces* A was most common (Fig. 6). In closed *M. michaelseni* mounds *Termitomyces* C was equally common in both small and large mounds. Unlike in open *M. subhyalinus* mounds, the internal temperature of *M. michaelseni* nests remained comparatively high throughout the year regardless of mound dimensions. When synthesizing all this information, it seems that *Termitomyces* species C only thrives in termite nests that remain very warm throughout the year. Optimal temperature for the growth of *Termitomyces* species is around 29–30 °C (Lüscher, 1961; Thomas, 1981). However, growth experiments with laboratory cultures (Thomas, 1981; A. Hakkarainen, unpublished, 2018) have revealed strain-specific differences in growth rates at suboptimal temperatures (20, 35, and 37 °C). Although experimental data comparing the thermal requirements of different *Termitomyces* species is currently scarse, our results suggest that *Termitomyces* species C does not perform optimally under the relatively low temperatures experienced in large *M. subhyalinus* mounds during the cool months of the year. Even if the fungal cultivations could tolerate low temperatures, their productivity may be seriously reduced during the cool period from June to August. This, in turn, may lead to an interruption in food supply that may weaken the termite colony and make it more vulnerable to predation etc. The presumed thermophilic nature of *Termitomyces* C may also be reflected in its overall distribution. The species seems to be restricted to the equatorial region, while *Termitomyces* A has a much wider overall range also covering the periodically cool savannas of South Africa (Rikkinen & Vesala, 2017; Vesala et al., 2017).

But why was *Termitomyces* C absent from large mounds of *M. subhyalinus* although it existed widely in small miniature mounds built by the same termite species? We can think of at least four factors that could play a role in explaining this phenomenon. The simple explanation would be that the initial selection of *Termitomyces* species C is facilitated by high temperatures. In other words the hot conditions of small *M. subhyalinus* mounds could give this fungus a competitive advantage, whereas the cooler temperatures of large mounds would favor *Termitomyces* species A and B. However, the initial choice of the fungal symbiont is believed to take place very early during colony formation, probably much before mound building starts (Nobre & Aanen, 2012). It also seems very unlikely that the symbiotic species could be subsequently changed during colony growth.

One must also consider the possibility that all *M. subhyalinus* mounds with *Termitomyces* C we sampled could have been young actively growing colonies and this would have accounted for their small size. This could mean that *M. subhyalinus* colonies cultivating *Termitomyces* C in our study area might largely be eliminated when their mounds grow over a critical limit and nest temperatures drop too low. However, *Macrotermes* mounds tend to grow fast during their first few years after which the growth rate slows down (Pomeroy, 1976). We followed three miniature mounds with *Termitomyces* C for over 4 years and did not notice any change in their dimensions. This clearly indicates that while these termite colonies were highly active, the mounds had reached some sort of developmental stasis.

A third possibility is that there exist natural variation in the mound building behavior of *M. subhyalinus* with some colonies just building large mounds and others miniature mounds. Presuming that the initial selection of symbiotic fungi is random, different combinations of the three *Termitomyces* species and different-sized mounds would then be expected. Assuming that temperatures in large mounds are occasionally too low for *Termitomyces* C all such combinations would thus become eliminated sooner or later. As the limited gas-exchange capacity of miniature mounds, however, must be disadvantageous for the termites, one can ask, what are the advantages of building such mounds?

As the fourth potential explanation we suggest that the termites actively adjust their mound building behavior to meet the specific thermal needs of their fungal symbionts. Host colonies would thus be able to monitor and respond to the growth performance of their fungal symbionts. As a result, the *M. subhyalinus* hosts of *Termitomyces* C occurring in our study area would refrain from building large mounds with effective ventilation systems as this would prevent them from maintaining the high temperature regime required by their fungal symbiont. As a negative trade-off the limited ventilation capacity of the reduced mounds could easily lead to increased CO_2_ levels in the underground nests that may limit the colony growth. A somewhat similar trade-off between temperature maintenance and gas-exchange has been previously documented for *M. bellicosus* in the Ivory Coast where termite mounds in cool gallery forests had thicker walls and reduced surface complexity compared to those in open savannas (Korb & Linsenmair, 1998a, 1999a; Korb, 2003).

## Conclusions

Fungus-growing termites of the genus *Macrotermes* cultivate several different *Termitomyces* species. Differences in the basic biology of the fungal symbionts are poorly known and the evolutionary forces behind the present diversity remain obscure. Our results suggest that nest temperatures are important in this context. Different *Termitomyces* species were found to be cultivated in small and large termite mounds which, in turn, had highly different internal temperatures especially during the coolest months of the year. Further studies are needed to explain these findings. However, it seems possible that the initial selection of a fungal symbiont by the newly established termite colony may affect the later mound building behavior of the insects.

## Supplemental Information

10.7717/peerj.6237/supp-1 Supplemental Information 1 All colonies included in this study and GenBank accession numbers for the published DNA sequences. All COI and most ITS sequences were produced in Vesala et al., 2017. New sequences produced in this study are indicated with an asterisk (*). In case of colonies where reference for COI is not given *Macrotermes* species was identified based on mound structure (open or closed ventilation). In case of those colonies where complete ITS sequences have not been published *Termitomyces* species was identified based on the two polymorphic sites in ITS1 region (see Vesala et al., 2017).

10.7717/peerj.6237/supp-2 Supplemental Information 2 Results of the statistical analysis.

10.7717/peerj.6237/supp-3 Supplemental Information 3 *Macrotermes subhyalinus* mounds in category ‘miniature’ (A–C) and ‘large’ (D–F). A: TR182, B: TR09, C: TR184, D: TM10, E: TR101, F: TR10. Length of the scale bar in each image is 45 cm and the diameter of the spherical targets (golf balls) is 4.2 cm. Photos by Risto Vesala.

10.7717/peerj.6237/supp-4 Supplemental Information 4 Air temperatures at study sites. Simultaneously measured diurnal mean temperatures at two study sites (Kasigau Road and Maktau) and at the weather station during 7th March 2016–26th October 2016.

10.7717/peerj.6237/supp-5 Supplemental Information 5 Temperatures in open and closed mounds. Simultaneously measured temperatures (24 measurements per day) in fungal chambers of two open (TS14, TS56) and two closed (TS18, TS19) termite mounds and the ambient air at Salt Lick study site during four months (Feb, Mar, Jun, Jul) in 2015.

10.7717/peerj.6237/supp-6 Supplemental Information 6 Temperature and architectural data from *M. subhyalinus* mounds. Diurnal mean temperatures (fungus chambers) and architectural parameters of *Macrotermes subhyalinus* mounds studied during the second measurement campaign (1st March 2016–31st March 2017) and simultaneous mean temperatures measured at the weather station. Diurnal mean temperatures and standard deviations are calculated from values registered on every three hours (eight measurements per each day).

10.7717/peerj.6237/supp-7 Supplemental Information 7 Mound size and *Termitomyces* species. Mound dimensions (height, mean basal width), ventilation types (open or closed) and cultivated *Termitomyces* species (A, B or C) of 164 termite colonies.

10.7717/peerj.6237/supp-8 Supplemental Information 8 Termitomyces ITS sequences.

## Additional Information and Declarations

### Field Study Permissions

The following information was supplied relating to field study approvals (i.e., approving body and any reference numbers): Field work was done under the research authorization from National Commission for Science, Technology and Innovation of Kenya (NACOSTI/P/17/54522/15694).

### DNA Deposition

The following information was supplied regarding the deposition of DNA sequences: New *Termitomyces* ITS sequences produced in this study have been deposited in GenBank (accession numbers MK275596–MK275616).

### Data Availability

The following information was supplied regarding data availability: The raw data are provided in the Supplemental Files.
