# Supplementary material for: Termite mound architecture regulates nest temperature and correlates with species identities of symbiotic fungi
Source: PeerJ. 2019 Jan 16;6:e6237. doi: 10.7717/peerj.6237 (PMC6339472; doi:10.7717/peerj.6237)
Supplement: Supplemental Information 1 — All COI and most ITS sequences were produced in Vesala et al., 2017. New sequences produced in this study are indicated with an asterisk (*). In case of colonies where reference for COI is not given Macrotermes species was identified based on mound structure (open or closed ventilation). In case of those colonies where complete ITS sequences have not been published Termitomyces species was identified based on the two polymorphic sites in ITS1 region (see Vesala et al., 2017). [file peerj-07-6237-s001.docx]

**Table S1.** All colonies included in this study and GenBank accession numbers for the published DNA sequences. All COI and most ITS sequences have been produced during our previous studies (Vesala et al. 2017). New sequences produced in this study are indicated with asterisk (*). In case of colonies where reference for COI is not given *Macrotermes* species was identified based on mound structure (open or closed ventilation). In case of those colonies where complete ITS sequences have not been published *Termitomyces* species was identified based on the two polymorphic sites within ITS1 region (see Vesala et al. 2017).

|  |  |  |  |  | **GenBank accession number** | |
| --- | --- | --- | --- | --- | --- | --- |
| **Colony** | **Study site** | ***Termitomyces***  **sp.** | ***Macrotermes* sp.** |  | ***Macrotermes* (COI)** | ***Termitomyces* (ITS)** |
| TK01 | Bungule | A | subhyalinus |  | KY197485 | KY197626 |
| TK02 | Bungule | A | subhyalinus |  | KY197486 |  |
| TK04 | Bungule | A | subhyalinus |  | KY197487 |  |
| TK05 | Bungule | A | subhyalinus |  | KY197488 |  |
| TK06 | Bungule | A | subhyalinus |  | KY197489 | KY197627 |
| TK07 | Bungule | A | subhyalinus |  | KY197490 |  |
| TK08 | Bungule | A | subhyalinus |  | KY197491 | KY197628 |
| TK09 | Bungule | A | subhyalinus |  | KY197492 | KY197629 |
| TK10 | Bungule | A | subhyalinus |  | KY197493 | KY197630 |
| TK11 | Bungule | A | subhyalinus |  | KY197494 |  |
| TK12 | Bungule | A | subhyalinus |  | KY197495 | KY197631 |
| TK13 | Bungule | A | subhyalinus |  | KY197496 | KY197632 |
| TK14 | Bungule | A | subhyalinus |  | KY197497 | KY197633 |
| TK15 | Bungule | A | subhyalinus |  | KY197498 |  |
| TK16 | Bungule | A | subhyalinus |  | KY197499 |  |
| TK17 | Bungule | A | subhyalinus |  | KY197500 | KY197634 |
| TK18 | Bungule | A | subhyalinus |  | KY197501 | KY197635 |
| TK19 | Bungule | A | subhyalinus |  | KY197502 | KY197636 |
| TK20 | Bungule | A | subhyalinus |  | KY197503 |  |
| TK21 | Bungule | A | subhyalinus |  | KY197504 | KY197637 |
| TK22 | Bungule | A | subhyalinus |  | KY197505 |  |
| TK23 | Bungule | A | subhyalinus |  | KY197506 |  |
| K1 | Kasigau Road | C | subhyalinus |  |  | MK275596* |
| TR83 | Kasigau Road | A | subhyalinus |  |  | KY197639 |
| TR09 | Kasigau Road | C | subhyalinus |  | KY197507 | KY197693 |
| TR10 | Kasigau Road | A | subhyalinus |  | KY197508 | KY197638 |
| TR101 | Kasigau Road | A | subhyalinus |  |  | MK275608* |
| TR109 | Kasigau Road | A | subhyalinus |  |  |  |
| TR149 | Kasigau Road | A | michaelseni |  | KY197607 | KY197640 |
| TR154 | Kasigau Road | A | subhyalinus |  | KY197509 | KY197641 |
| TR156 | Kasigau Road | A | subhyalinus |  | KY197510 |  |
| TR159 | Kasigau Road | A | michaelseni |  | KY197609 | KY197642 |
| TR160 | Kasigau Road | A | michaelseni |  | KY197610 |  |
| TR161 | Kasigau Road | A | subhyalinus |  | KY197511 | KY197643 |
| TR164 | Kasigau Road | A | michaelseni |  | KY197611 | KY197644 |
| TR166 | Kasigau Road | C | michaelseni |  | KY197612 | KY197694 |
| TR167 | Kasigau Road | A | subhyalinus |  | KY197512 |  |
| TR168 | Kasigau Road | C | subhyalinus |  | KY197513 | KY197695 |
| TR172 | Kasigau Road | C | subhyalinus |  | KY197514 | KY197696 |
| TR173 | Kasigau Road | A | michaelseni |  | KY197613 |  |
| TR175 | Kasigau Road | C | michaelseni |  | KY197614 | KY197697 |
| TR182 | Kasigau Road | A | subhyalinus |  |  | MK275609* |
| TR183 | Kasigau Road | C | subhyalinus |  |  | MK275610* |
| TR184 | Kasigau Road | A | subhyalinus |  |  |  |
| TR185 | Kasigau Road | A | subhyalinus |  |  |  |
| TT01 | Latika | C | subhyalinus |  |  | MK275615* |
| TT02 | Latika | A | subhyalinus |  |  |  |
| TL01 | Lions Bluff | A | subhyalinus |  |  | MK275599* |
| TL02 | Lions Bluff | A | subhyalinus |  |  |  |
| TL05 | Lions Bluff | C | subhyalinus |  |  | MK275600* |
| TL06 | Lions Bluff | A | subhyalinus |  |  |  |
| TL07 | Lions Bluff | A | subhyalinus |  |  |  |
| TL08 | Lions Bluff | A | subhyalinus |  |  |  |
| TL09 | Lions Bluff | C | michaelseni |  |  | MK275601* |
| TL10 | Lions Bluff | A | michaelseni |  |  |  |
| TL11 | Lions Bluff | A | michaelseni |  |  | MK275602* |
| TL12 | Lions Bluff | C | michaelseni |  |  | MK275603* |
| TL13 | Lions Bluff | A | michaelseni |  |  |  |
| TL14 | Lions Bluff | C | michaelseni |  |  | MK275604* |
| TL15 | Lions Bluff | C | subhyalinus |  |  | MK275605* |
| TL16 | Lions Bluff | C | subhyalinus |  |  | MK275606* |
| TL17 | Lions Bluff | A | subhyalinus |  |  |  |
| TL18 | Lions Bluff | A | subhyalinus |  |  | MK275607* |
| TD01 | Maktau | A | subhyalinus |  |  |  |
| TM02 | Maktau | A | subhyalinus |  | KY197516 | KY197645 |
| TM04 | Maktau | B | subhyalinus |  | KY197517 | KY197687 |
| TM07 | Maktau | B | subhyalinus |  | KY197520 |  |
| TM08 | Maktau | C | subhyalinus |  | KY197521 | KY197698 |
| TM10 | Maktau | A | subhyalinus |  | KY197522 | KY197646 |
| TM14 | Maktau | A | subhyalinus |  | KY197523 | KY197647 |
| TM15 | Maktau | A | subhyalinus |  | KY197524 | KY197648 |
| TM16 | Maktau | A | subhyalinus |  | KY197525 | KY197649 |
| TM19 | Maktau | B | subhyalinus |  | KY197527 | KY197688 |
| TM23 | Maktau | A | subhyalinus |  | KY197529 | KY197650 |
| TM25 | Maktau | A | subhyalinus |  | KY197530 | KY197651 |
| TM26 | Maktau | A | subhyalinus |  | KY197531 | KY197652 |
| TM33 | Maktau | A | subhyalinus |  | KY197533 | KY197653 |
| TM35 | Maktau | B | subhyalinus |  | KY197535 | KY197689 |
| TM36 | Maktau | C | subhyalinus |  | KY197536 | KY197699 |
| TM37 | Maktau | C | subhyalinus |  | KY197537 | KY197700 |
| TM39 | Maktau | B | subhyalinus |  | KY197539 | KY197690 |
| TM41 | Maktau | B | subhyalinus |  | KY197540 | KY197691 |
| TM42 | Maktau | A | subhyalinus |  | KY197541 | KY197654 |
| MR1 | Mbula | C | michaelseni |  |  |  |
| MR2 | Mbula | A | subhyalinus |  |  |  |
| MR4 | Mbula | A | subhyalinus |  |  |  |
| MR5 | Mbula | A | subhyalinus |  |  |  |
| MR6 | Mbula | C | subhyalinus |  |  |  |
| TB02 | Mbula | B | subhyalinus |  | KY197542 |  |
| TB03 | Mbula | A | subhyalinus |  | KY197543 |  |
| TB04 | Mbula | C | subhyalinus |  | KY197544 | KY197701 |
| TB06 | Mbula | A | subhyalinus |  | KY197546 |  |
| TB07 | Mbula | A | subhyalinus |  | KY197547 |  |
| TB08 | Mbula | A | michaelseni |  | KY197615 |  |
| TB10 | Mbula | C | subhyalinus |  |  | KY197705 |
| TB26 | Mbula | A | subhyalinus |  | KY197555 | KY197655 |
| TB33 | Mbula | A | subhyalinus |  | KY197556 | KY197656 |
| TB35 | Mbula | A | subhyalinus |  | KY197557 |  |
| TB36 | Mbula | A | subhyalinus |  | KY197558 |  |
| TA01 | Mgeno | A | subhyalinus |  | KY197574 | KY197657 |
| TA02 | Mgeno | B | subhyalinus |  | KY197575 | KY197692 |
| TA05 | Mgeno | A | subhyalinus |  | KY197576 | KY197658 |
| TA06 | Mgeno | A | subhyalinus |  | KY197577 | KY197659 |
| TA11 | Mgeno | A | subhyalinus |  | KY197578 | KY197660 |
| TA12 | Mgeno | A | subhyalinus |  | KY197579 | KY197661 |
| TA19 | Mgeno | A | subhyalinus |  | KY197580 | KY197662 |
| TA21 | Mgeno | A | subhyalinus |  | KY197581 | KY197663 |
| TA26 | Mgeno | A | subhyalinus |  | KY197583 | KY197664 |
| TA29 | Mgeno | C | subhyalinus |  | KY197584 | KY197702 |
| TA30 | Mgeno | A | subhyalinus |  | KY197585 |  |
| TA31 | Mgeno | A | subhyalinus |  | KY197586 | KY197665 |
| TA32 | Mgeno | A | subhyalinus |  | KY197587 | KY197666 |
| TA36 | Mgeno | A | subhyalinus |  | KY197588 |  |
| TY01 | Mgeno | A | subhyalinus |  | KY197589 | KY197667 |
| TY02 | Mgeno | A | subhyalinus |  | KY197590 | KY197668 |
| TY04 | Mgeno | C | subhyalinus |  | KY197591 | KY197703 |
| TY06 | Mgeno | A | subhyalinus |  | KY197592 | KY197669 |
| TY09 | Mgeno | C | subhyalinus |  | KY197593 | KY197704 |
| TY11 | Mgeno | A | subhyalinus |  | KY197594 |  |
| TY12 | Mgeno | A | subhyalinus |  | KY197595 | KY197670 |
| TY13 | Mgeno | A | subhyalinus |  | KY197596 |  |
| TY14 | Mgeno | A | subhyalinus |  | KY197597 | KY197671 |
| TY17 | Mgeno | A | subhyalinus |  | KY197598 | KY197672 |
| TFA11 | Mwashoti | A | subhyalinus |  | KY197560 |  |
| TFA12 | Mwashoti | A | subhyalinus |  | KY197561 | KY197673 |
| TFA13 | Mwashoti | A | subhyalinus |  | KY197562 |  |
| TFA14 | Mwashoti | A | subhyalinus |  | KY197563 | KY197674 |
| TFA18 | Mwashoti | A | subhyalinus |  | KY197564 |  |
| TFA21 | Mwashoti | A | subhyalinus |  | KY197565 |  |
| TFA28 | Mwashoti | A | subhyalinus |  | KY197566 |  |
| TFA35 | Mwashoti | C | subhyalinus |  | KY197567 | KY197706 |
| TFA48 | Mwashoti | A | subhyalinus |  | KY197569 | KY197675 |
| TFA49 | Mwashoti | A | subhyalinus |  | KY197570 |  |
| TFB20 | Mwashoti | A | subhyalinus |  | KY197571 |  |
| TFB34 | Mwashoti | A | subhyalinus |  | KY197572 |  |
| TFB50 | Mwashoti | A | subhyalinus |  | KY197573 |  |
| TU01 | Mwashuma | C | michaelseni |  |  | MK275616* |
| TU02 | Mwashuma | A | michaelseni |  |  |  |
| S1 | Salt Lick | C | michaelseni |  |  | MK275597* |
| S5 | Salt Lick | C | subhyalinus |  |  | MK275598* |
| TS08 | Salt Lick | A | subhyalinus |  | KY197600 | KY197679 |
| TS09 | Salt Lick | A | subhyalinus |  | KY197601 | KY197680 |
| TS10 | Salt Lick | A | subhyalinus |  | KY197602 | KY197681 |
| TS12 | Salt Lick | A | subhyalinus |  | KY197620 | KY197682 |
| TS13 | Salt Lick | A | subhyalinus |  | KY197603 | KY197683 |
| TS14 | Salt Lick | A | subhyalinus |  | KY197604 | KY197684 |
| TS15 | Salt Lick | A | michaelseni |  | KY197621 | KY197685 |
| TS16 | Salt Lick | C | michaelseni |  | KY197622 | KY197708 |
| TS18 | Salt Lick | A | michaelseni |  | KY197623 |  |
| TS19 | Salt Lick | A | michaelseni |  | KY197624 |  |
| TS20 | Salt Lick | A | subhyalinus |  | KY197605 |  |
| TS22 | Salt Lick | C | michaelseni |  | KY197625 | KY197707 |
| TS56 | Salt Lick | A | subhyalinus |  | KY197606 |  |
| TS200 | Salt Lick | A | subhyalinus |  |  |  |
| TS201 | Salt Lick | A | subhyalinus |  |  | MK275611* |
| TS202 | Salt Lick | C | subhyalinus |  |  | MK275612* |
| TS203 | Salt Lick | A | subhyalinus |  |  |  |
| TS204 | Salt Lick | A | subhyalinus |  |  |  |
| TS205 | Salt Lick | A | subhyalinus |  |  |  |
| TS206 | Salt Lick | A | subhyalinus |  |  |  |
| TS207 | Salt Lick | C | subhyalinus |  |  | MK275613* |
| TS208 | Salt Lick | A | subhyalinus |  |  | MK275614* |
| TH01 | Sasha Camp | C | subhyalinus |  |  |  |
| TH04 | Sasha Camp | A | subhyalinus |  |  |  |
