# Supplementary material for: Termite mound architecture regulates nest temperature and correlates with species identities of symbiotic fungi
Source: PeerJ. 2019 Jan 16;6:e6237. doi: 10.7717/peerj.6237 (PMC6339472; doi:10.7717/peerj.6237)
Supplement: Supplemental Information 2 [file peerj-07-6237-s002.docx]

**Supplementary Table S2.** Comparison of the effects of different architectural variables on fungus chamber temperatures in studied *M. subhyalinus* mounds during A) warmest (March) and B) coolest (July) months of the year. Each model contains ambient air temperature (mean of the previous day at the weather station) and one architectural variable as fixed variables. L.Ratio and p-values calculated from pairwise comparisons with the initial model having ambient air temperature as the only fixed variable.

A. March

| **Fixed effects:** |  |  |  |  |
| --- | --- | --- | --- | --- |
| **Nest temp. ~ ambient air temp. +** | **AIC** | **∆_AIC_** | **L.Ratio** | **p-value** |
| Mound height | 349.322 | 12.853 | 8.92 | 0.0028 |
| Mound width | 345.316 | 8.847 | 12.92 | <0.001 |
| Mound volume | 352.402 | 15.933 | 5.84 | 0.0157 |
| Mound surface area | 347.893 | 11.424 | 10.35 | 0.0013 |
| Openings maximum distance | 341.996 | 5.527 | 16.25 | <0.001 |
| Mound type | 336.469 | 0 | 21.77 | <0.0001 |

B. July

| **Fixed effects:** |  |  |  |  |
| --- | --- | --- | --- | --- |
| **Nest temp. ~ ambient air temp. +** | **AIC** | **∆_AIC_** | **L.Ratio** | **p-value** |
| Mound height | 300.077 | 13.309 | 11.27 | <0.001 |
| Mound width | 294.282 | 7.514 | 17.06 | <0.0001 |
| Mound volume | 302.882 | 16.114 | 8.46 | 0.0036 |
| Mound surface area | 298.274 | 11.506 | 13.07 | <0.001 |
| Openings maximum distance | 292.851 | 6.083 | 18.49 | <0.0001 |
| Mound type | 286.768 | 0 | 24.57 | <0.0001 |

**Supplementary Table S3.** Parameter estimates of the final GLS models including all the significant variables and their interactions.

A. March

|  | **Value** | **Std.Error** | **t-value** | **p-value** |
| --- | --- | --- | --- | --- |
| (Intercept) | 22.019674 | 1.107602 | 19.880494 | 0 |
| Ambient air temperature | 0.292806 | 0.0432727 | 6.766547 | 0 |
| Type_miniature | 3.43067 | 1.0687266 | 3.210054 | 0.0014 |
| Site_KasigauRoad | 3.91123 | 1.2167987 | 3.214361 | 0.0014 |
| Ambient air temperature : Type_miniature | -0.09372 | 0.0417538 | -2.244582 | 0.0254 |
| Ambient air temperature : Site_KasigauRoad | -0.152823 | 0.0475388 | -3.214708 | 0.0014 |

B. July

|  | **Value** | **Std.Error** | **t-value** | **p-value** |
| --- | --- | --- | --- | --- |
| (Intercept) | 21.340022 | 0.7479059 | 28.533032 | 0 |
| Ambient air temperature | 0.136863 | 0.0325398 | 4.206013 | 0 |
| Type_miniature | 2.959901 | 0.4379428 | 6.758647 | 0 |
| LAI | 1.253993 | 0.5649474 | 2.219664 | 0.0271 |

**Supplementary Table S4.** Results of the One-way ANOVAs comparing mound height and width in termite colonies cultivating different *Termitomyces* species (sp. A, B or C).

| **A. Open mounds: mound height ~ *Termitomyces* species** | |  |  |  |
| --- | --- | --- | --- | --- |
|  | **Estimate** | **Std.Error** | **t-value** | **p-value** |
| (Intercept) | 116.38 | 5.01 | 23.231 | <0.0001 |
| fungi_spB | 28.62 | 18.99 | 1.507 | 0.134267 |
| fungi_spC | -47.97 | 12.13 | -3.955 | 0.000124 |
|  |  |  |  |  |
|  |  |  |  |  |
| **B. Open mounds: mound mean width ~ *Termitomyces* species** | | |  |  |
|  | **Estimate** | **Std.Error** | **t-value** | **p-value** |
| (Intercept) | 297.542 | 8.443 | 35.24 | <0.0001 |
| fungi_spB | -18.792 | 32.013 | -0.587 | 0.558177 |
| fungi_spC | -76.883 | 20.446 | -3.76 | 0.000253 |
|  |  |  |  |  |
|  |  |  |  |  |
| **C. Closed mounds: mound height ~ Termitomyces species** | |  |  |  |
|  | **Estimate** | **Std.Error** | **t-value** | **p-value** |
| (Intercept) | 112.81 | 11.96 | 9.429 | <0.0001 |
| fungi_spC | -16.45 | 18.74 | -0.878 | 0.389 |
|  |  |  |  |  |
|  |  |  |  |  |
| **D. Closed mounds: mound mean width ~ Termitomyces species** | | |  |  |
|  | **Estimate** | **Std.Error** | **t-value** | **p-value** |
| (Intercept) | 293.59 | 31.08 | 9.445 | <0.0001 |
| fungi_spC | -13.46 | 48.7 | -0.276 | 0.785 |
