# Supplementary material for: Termite mound architecture regulates nest temperature and correlates with species identities of symbiotic fungi
Source: PeerJ. 2019 Jan 16;6:e6237. doi: 10.7717/peerj.6237 (PMC6339472; doi:10.7717/peerj.6237)
Supplement: Supplemental Information 3 — A: TR182, B: TR09, C: TR184, D: TM10, E: TR101, F: TR10. Length of the scale bar in each image is 45 cm and the diameter of the spherical targets (golf balls) is 4.2 cm. Photos by Risto Vesala. [file peerj-07-6237-s003.pdf]

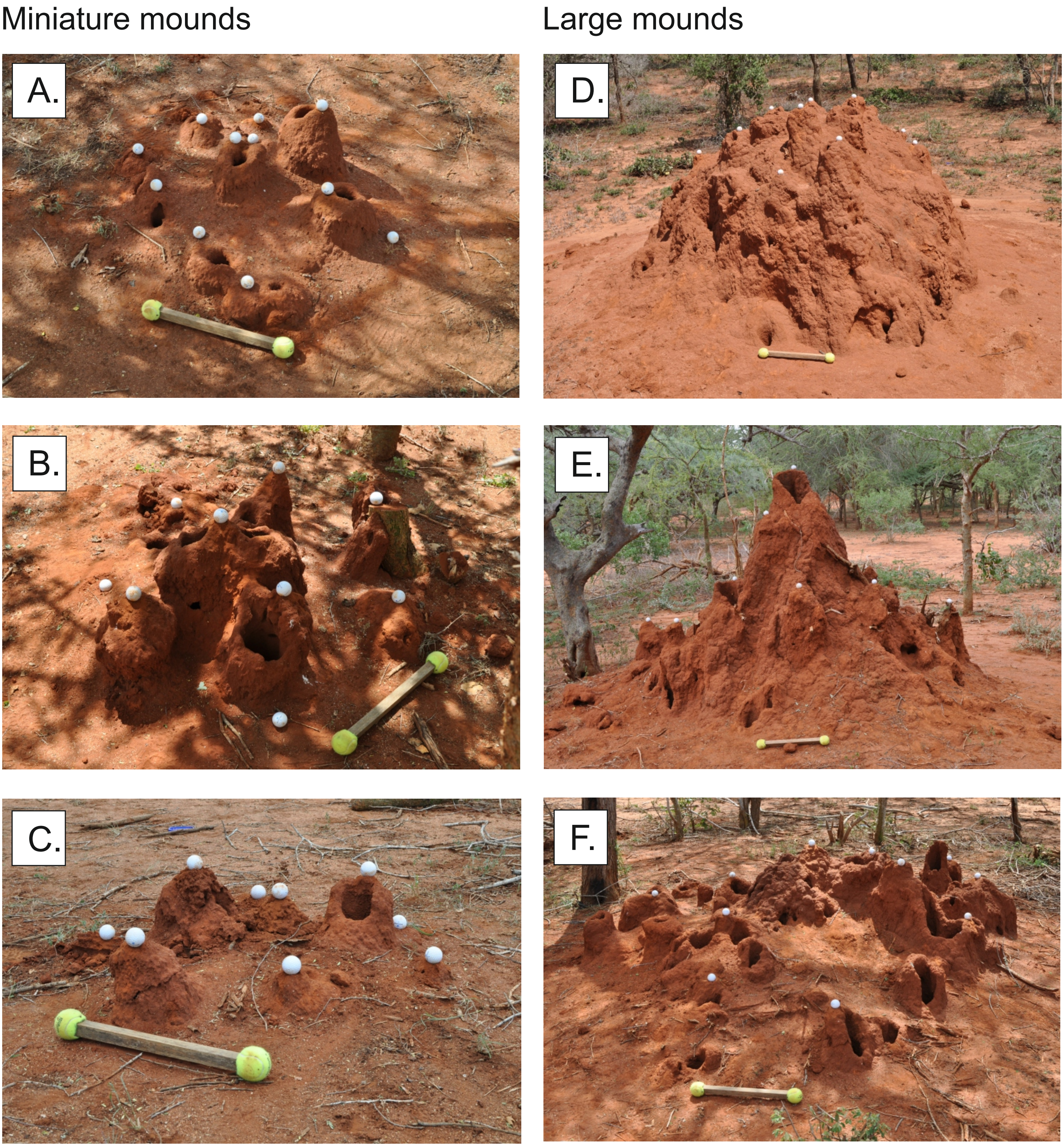

Supplementary Figure S1. *Macrotermes subhyalinus* mounds in category 'miniature' (A–C) and 'large' (D–F). A: TR182, B: TR09, C: TR184, D: TM10, E: TR101, F: TR10. Length of the scale bar in each image is 45 cm and the diameter of the spherical targets (golf balls) is 4.2 cm. Photos by Risto Vesala.
